# Oxygen/Nitric Oxide Dual-Releasing Nanozyme for Augmenting TMZ-Mediated Apoptosis and Necrosis

**DOI:** 10.1021/acs.molpharmaceut.4c00817

**Published:** 2024-11-21

**Authors:** Jun Ma, Jingjing Qiu, Gus A. Wright, Shiren Wang

**Affiliations:** † Department of Biomedical Engineering, Texas A&M University, College Station, Texas 77843, United States; ‡ Department of Mechanical Engineering & Department of Materials Science and Engineering, Texas A&M University, College Station, Texas 77843, United States; § Flow Cytometry Facility, College of Veterinary Medicine & Biomedical Sciences, Texas A&M University, College Station, Texas 77843, United States; ∥ Department of Industrial Systems and Engineering & Department of Materials Science and Engineering & Department of Biomedical Engineering, Texas A&M University, College Station, Texas 77843, United States

**Keywords:** nanoceria, cancer cell membrane, temozolomide, oxygen, nitric oxide, lysosome, reactive oxygen species, apoptosis and necrosis

## Abstract

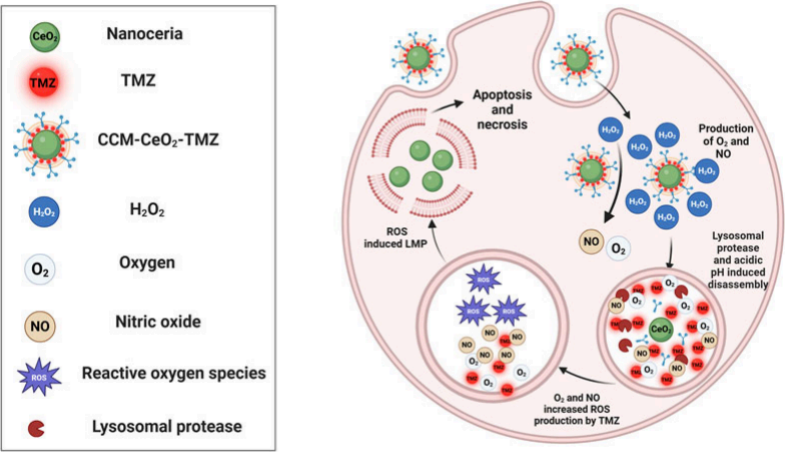

Glioblastoma multiforme (GBM) is the most common and aggressive malignant brain tumor, with a poor prognosis. Temozolomide (TMZ) represents the standard chemotherapy for GBM but has limited efficacy due to poor targeting and a hypoxic tumor microenvironment (TME). To address these challenges, we developed a dual-gas-releasing, cancer-cell-membrane-camouflaged nanoparticle to deliver TMZ. This nanoceria, camouflaged with a cancer cell membrane (CCM-CeO_2_), targets explicitly GBM cells and accumulates in lysosomes, triggering the rapid release of TMZ. Additionally, CCM-CeO_2_ could release oxygen (O_2_) and nitric oxide (NO) in response to the TME. Synthesized using d-arginine, catalytic nanoceria could decompose excessive hydrogen peroxide (H_2_O_2_) in the TME to produce O_2_, while d-arginine could nonenzymatically react with H_2_O_2_ to generate NO. CCM-CeO_2_ could penetrate GBM spheroids to a depth of 148.3 ± 31 μm, with the O_2_ and NO produced, reducing HIF-1α protein expression. When loaded with TMZ, CCM-CeO_2_ could increase the intracellular ROS produced by TMZ, leading to lysosome membrane permeabilization and notably augmented apoptosis and necrosis in GBM cells. An in vitro antitumor assay using spheroids showed that CCM-CeO_2_ reduced the IC_50_ value of TMZ from 174.5 to 42.6 μg/mL, likely due to the catalase-like activity of nanoceria. These results suggest that alleviating hypoxia and increasing ROS produced by chemotherapeutics could be an effective therapeutic strategy for treating GBM.

## Introduction

Glioblastoma multiforme (GBM) is the most malignant brain tumor, with an extremely low five-year survival rate of 6.8%^1^ and a mean survival time of less than 16 months.^2^ The standard treatment for newly diagnosed GBM includes surgical resection followed by radiotherapy and chemotherapy, primarily with TMZ.^3^ However, TMZ has shown limited efficacy as the first-line chemotherapy, increasing mean survival by only 2.5 to 4 months.^3,4^ While TMZ is somewhat able to cross the blood–brain barrier (BBB),^5^ less than 1% of the drug reaches the GBM^6^ due to its aqueous instability and lack of specific targeting. Increasing the dosage of TMZ leads to systemic toxicity without improving drug concentration in the GBM.^6^ Additionally, cellular and genetic resistance further limits the efficacy of TMZ therapy. TMZ is an alkylating agent that methylates DNA and produces cytotoxic O^6^-methylguanine (O^6^-MeG), which induces cancer cell apoptosis.^7^ However, over 60% of GBM tumors upregulate O^6^-methylguanine methyltransferase (MGMT) that can repair the DNA damage induced by O^6^-MeG,^8^ and MGMT has been an important indicator of responsiveness of patients to alkylating agents.^9−11^ Furthermore, hypoxia in the tumor microenvironment (TME) also contributes to the TMZ resistance. In adapting to low oxygen levels, hypoxia upregulates hypoxia-inducible factor-1 (HIF-1), promoting tumor proliferation, angiogenesis, invasion, and metastasis.^12^ In addition, cancer stem cells (CSCs) are enriched within the hypoxic GBM core and overexpress MGMT, highlighting the essential role of hypoxia in therapeutic resistance.^13^ On the other hand, TMZ combined with hyperbaric oxygen notably inhibits glioma cell growth,^14^ induces apoptosis,^15^ and enhances antitumor efficacy,^14,15^ indicating that alleviating hypoxia is essential for improving TMZ therapy.

In recent years, in situ O_2_ production within the tumors by catalytic decomposition of H_2_O_2_ has emerged as a promising strategy to alleviate hypoxia and potentiate chemotherapy. Catalase-mimetic materials, such as MnO_2_^16−18^ and Pt,^19,20^ have been widely explored for this purpose but face challenges, including rapid degradation and cytotoxicity. For instance, MnO_2_ degrades quickly in the presence of H_2_O_2_, acidic environments, and glutathione (GSH),^21^ losing continuous O_2_ production capacity in the TME^22^ and releasing toxic Mn^2+^ ions.^23^ Similarly, Pt-based nanocatalysts are unstable and prone to aggregation, leading to reduced catalytic activity.^19^ In contrast, cerium oxide (CeO_2_) nanoparticles exhibit a unique auto regenerative property which involves the recycling between Ce^3+^ and Ce^4+^,^24,25^ enabling sustained catalytic activities. The enzymatic activity of CeO_2_ is pH-dependent: it effectively generates O_2_ at physiological pH (7.4), while at acidic pH, it displays decreased catalase-like activity^26^ and shifts to enhanced superoxide dismutase (SOD)-mimetic and oxidase activities, scavenging superoxide ions, accumulating H_2_O_2_,^27^ and producing reactive oxygen species (ROS).^28^ The pH-responsive behaviors of CeO_2_ have shown promise in drug delivery systems, leveraging its unique oxidase activity.^29−31^ However, hypoxic TME limits its O_2_-dependent oxidase activity, questioning the mechanism of CeO_2_-enhanced chemotherapy and suggesting an alternative approach to alleviating hypoxia. The external supply of nitric oxide (NO) can counteract hypoxia-induced chemoresistance,.^32,33^ as low NO levels degrade HIF-1α, increase intracellular oxygen availability,^34^ and sensitize chemotherapy by inducing mitochondrial tyrosine nitration and apoptosis.^35^ However, high NO levels can be cytotoxic^36^ and induce cell arrest/cell death,^37^ highlighting the importance of controlled delivery of NO to cancer cells to boost chemotherapy while minimizing the side effects. While O_2_^14,15,38−40^ or NO^32,35,41−45^ alone have been widely used to enhance chemotherapy, the dual, TME-responsive release of both gases remains largely unexplored.

**Scheme 1:**
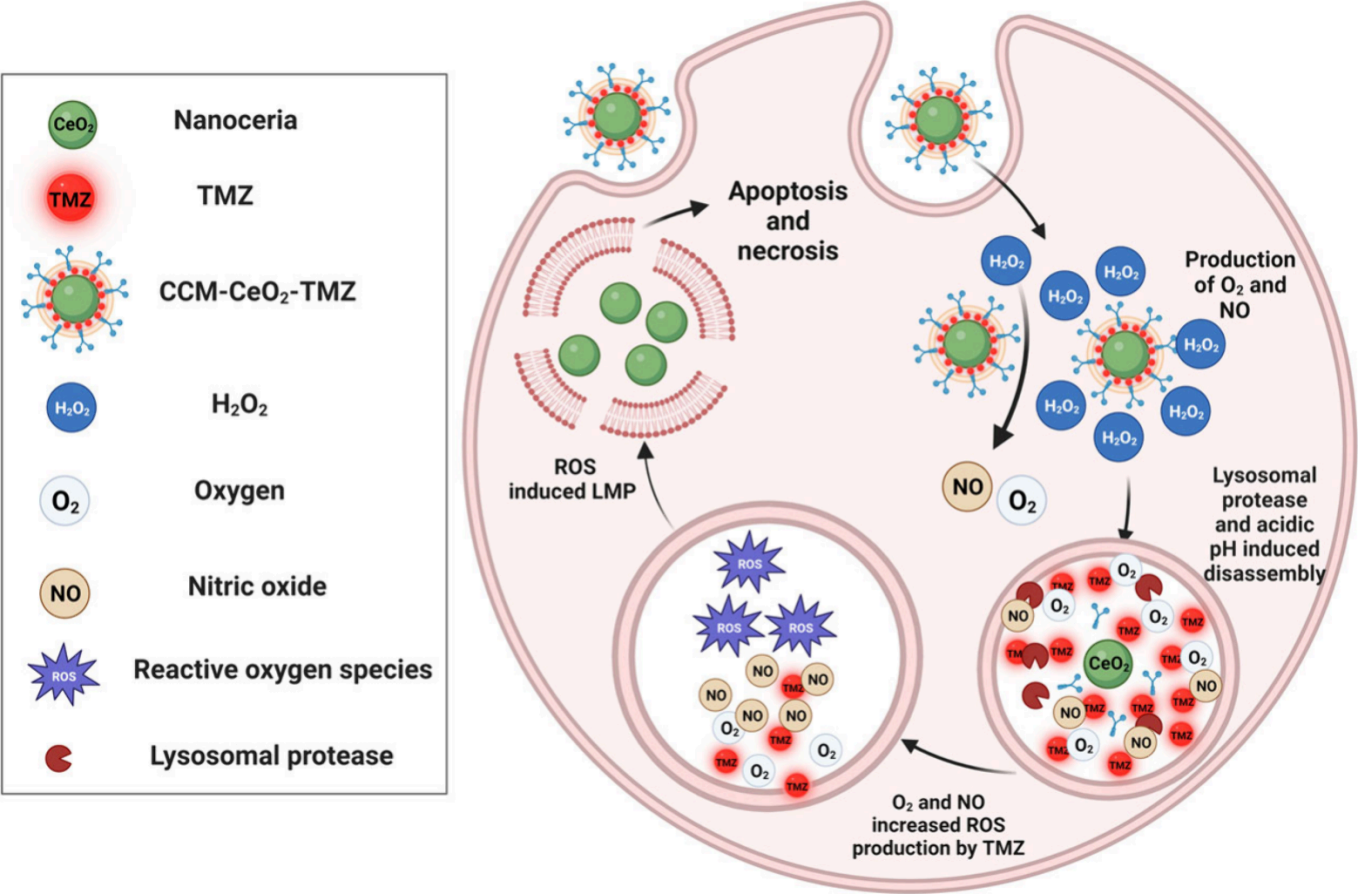
Mechanism of Augmented TMZ-Induced Apoptosis and Necrosis by CCM-CeO_2_ Once internalized, CCM-CeO_2_ decomposes H_2_O_2_ and nonenzymatically reacts with H_2_O_2_ to produce NO and O_2_ in the cytoplasm, and then CCM-CeO_2_-TMZ preferentially accumulates in lysosomes. Lysosomal proteases and acidic pH facilitate the rapid TMZ release. NO and O_2_ can alleviate hypoxia and increase ROS produced by TMZ, which damages the lysosome membrane, leading to cancer cell apoptosis and necrosis. Produced with permission from Biorender.

To address the above challenges, we engineered GBM cell-membrane-camouflaged nanoceria to target GBM and alleviate hypoxia by producing O_2_ and NO in situ (Scheme 1). Inspired by the ability of circulating tumor cells to colonize their tumors of origin,^46^ cancer cell membranes (CCMs) have been extensively explored as self-targeting materials. Brain metastatic tumor cell membrane^47^ and GBM cell membrane^48^ have been directly used as coating materials for drug carriers. Thus, the CCM could facilitate the targeting of CeO_2_ by GBM with high efficiency in our design. The simultaneous release of O_2_ and NO could be triggered by excessive H_2_O_2_ in the TME. After internalization, CCM-CeO_2_ could accumulate in lysosomes, triggering the rapid release of loaded TMZ. In addition, lysosomes in cancer cells lack antioxidant enzymes^49,50^ and are more vulnerable to oxidative stress.^51^ In light of this property, as-produced O_2_ and NO could increase ROS production by rapidly releasing TMZ in lysosomes, which could, in turn, damage lysosome integrity and further augment apoptosis and necrosis.

## Experimental Section

### Materials

Ce(NO_3_)_3_·6H_2_O, d-arginine, 200 proof ethanol, temozolomide (TMZ), SIGMAFAST protease inhibitor cocktail tablets, 4,5-diamino-rhodamine B (DAR-1), and acridine orange solution were purchased from Sigma-Aldrich (St. Louis, MO). The ultrasmall CeO_2_ (<5 nm) was obtained from Alfa Aesar and used as a control for FTIR analysis. Human glioblastoma cell line U-87 MG and Eagle’s minimum essential medium (EMEM) were purchased from ATCC (Manassas, VA). Fetal bovine serum (FBS), penicillin/streptomycin solution, 0.05% EDTA–trypsin, Dulbecco’s phosphate-buffered saline (DPBS), a Live/Dead cell imaging kit, a CellROX reagent variety pack, and Image-iT Green hypoxia reagent were obtained from Thermo Fisher Scientific (Waltham, MA). Human brain microvascular endothelial cells (HBMECs) and an endothelial cell medium kit were obtained from ScienCell (Carlsbad, CA). Lysobrite green, Alexa Fluor 594-HIF, and a Cell Meter Apoptotic and Necrotic Multiplexing Detection Kit were purchased from ATT Bioquest (Pleasanton, CA).

### Cell Culture

U-87 cells were cultured in EMEM supplemented with 10% FBS and 1% penicillin/streptomycin at 37 °C, 5% CO_2_, and 95% relative humidity until ∼90% confluence was reached. Then, cells were harvested with 0.05% EDTA–trypsin and used for further assays.

For HBMEC culture, the cell culture flask was first coated with 1% gelatin/fibronectin solution in a 37 °C incubator overnight, and then the flask was washed with DPBS three times. HBMECs were cultured in an endothelial cell medium supplemented with 5% FBS and 1% endothelial cell growth supplement. Similarly, after ∼90% confluence, the cells were harvested by 0.05% EDTA–trypsin for further use.

### Synthesis and Characterizations of Nanoparticles

Nanoceria (CeO_2_) was synthesized by alkaline precipitation. Ce(NO_3_)_3_·6H_2_O was dissolved in 200 proof ethanol to prepare 200 mM solution, and then 100 mM d-arginine solution was prepared in deionized (DI) water. Next, 0.5 mL of 200 mM Ce(NO_3_)_3_ was added to 7.5 mL of ethanol under stirring. Then, 2 mL of 100 mM d-arginine solution was added dropwise to the solution under stirring. The mixture solution was stirred for 1 h, and as-synthesized CeO_2_ was washed with ethanol and DI water.

U-87 cells were harvested and washed with 4 °C DPBS three times. Then cells were resuspended in hypotonic lysing buffer (SIGMAFAST protease inhibitor tablet, 20 mM Tris-HCl pH 7.5, 10 mM KCl, 2 mM MgCl_2_) and kept in an ice bath for 15 min. Afterward, the CCM solution was frozen at −80 °C for at least 30 min and then the solution was thawed at room temperature. After three freeze–thaw cycles, the CCM solution was centrifuged at 700 g for 10 min. The pellet was discarded, and the supernatant was centrifuged at 14,000 g for 30 min. The cell pellets were collected, resuspended in DI water, and stored at 4 °C for further use. The protein concentration of CCM was quantified with Bradford reagent (Biorad, CA).

To prepare CCM-coated CeO_2_ (CCM-CeO_2_), 600 μg of CeO_2_ was mixed with 200 μg of CCM solution and ultrasonicated on ice for 90 s (15 s on, 15 s off, 120 W, 50% amplitude, Fisher Scientific, Model 120). After sonication, CCM-CeO_2_ was collected by centrifugation for further use. To load TMZ, 1200 μg of TMZ (dissolved in DMSO) was added to CCM-CeO_2_, ultrasonicated for 90 s, and collected by centrifugation. The supernatant was collected, and TMZ concentration was analyzed by a Nanodrop One instrument at an absorbance wavelength of 328 nm. The standard curve of TMZ at 328 nm was also prepared using the Nanodrop One instrument, and based on the standard curve, the drug loading efficiency (LE) and loading capacity (LC) were determined by the following equations:
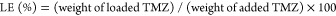

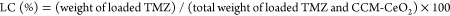


For transmission electronic microscopy (TEM) characterization, 5 μL of nanoparticle solution in DI water was drop-cast onto a TEM grid. After 10 min, the remaining solution was removed with a filter paper. For the negative staining of CCM-CeO_2_, after removing the remaining nanoparticle solution, 5 μL of 1% phosphotungstic acid (neutral pH, adjusted with NaOH) was applied to the TEM grid. After another 10 min, the remaining stain was removed with filter paper. The samples were left to dry at room temperature overnight, and TEM images were taken by using an FEI Morgagni 260 instrument. CCM-CeO_2_ was also imaged with atomic force microscopy (AFM, Bruker MultiMode 8-HR). The valence state of Ce ions on the CeO_2_ surface was also detected by X-ray photoelectron spectroscopy (XPS, Omicron ESCA) and UV–vis scanning (Hitachi U-4100). The presence of d-arginine on d-CeO_2_ was analyzed by a Fourier transform infrared spectrometer (FTIR, Burker ALPHA-Platinum).

### Enzymatic Activity Assays

The SOD activity of CCM-CeO_2_ was detected by a SOD Assay Kit (Thermo Fisher), according to the manufacturer’s protocol. For oxidase activity, 100 μg/mL CCM-CeO_2_ was mixed with 0.4 g/L TMB solution, and the absorbance at 652 nm was monitored at different time points. For peroxidase activity, 100 μg/mL of CCM-CeO_2_ was mixed with 100 μM H_2_O_2_ and 10 mg/mL *o*-phenylenediamine (OPD). The absorbance at 417 nm was monitored using a microplate reader (Biotek Cytation 5).

### O_2_ and NO Production Capacity of CCM-CeO_2_ in Aqueous Solution

First, 100 μg/mL CCM-CeO_2_ in DPBS was mixed with 100 μM H_2_O_2_, and at specific time points, samples were taken and analyzed for dissolved oxygen. The dissolved oxygen level was measured by a Pro20 dissolved oxygen meter (YSI) with DPBS as a control. The produced NO was detected by 5 μM 4,5-diamino-rhodamine B (DAR-1, Sigma-Aldrich), and the fluorescence intensity was monitored by a microplate reader (Biotek Cytation 5) with an excitation wavelength at 566 nm and an emission wavelength at 586 nm. Then an Amplex Red Hydrogen Peroxide/Peroxidase Assay Kit (Thermo Fisher) was used to detect the amount of H_2_O_2_ consumed by CCM-CeO_2_.

### Cell Viability Assay

To form spheroids with a diameter >400 μm, 10,000 U-87 cells were seeded in a U-bottom ultralow attachment 96-well plate (S-bio) and incubated for 3 days. Then, the spheroids were treated with various concentrations of CeO_2_ and CCM-CeO_2_ for 7 days (0–800 μg/mL). Afterward, according to the manufacturer’s protocol, 1× Calcein AM was added to each well and incubated at room temperature for 1 h. Subsequently, the fluorescence intensity was measured by a microplate reader (Cytation 5, Biotek) with an excitation wavelength of 488 nm and an emission wavelength of 515 nm. The cell viability of treated spheroids was normalized to untreated spheroids.

### Homotypic Targeting Assay

One milligram of d-CeO_2_ was conjugated with 10 μg of Alexa Fluor 488-NHS ester (AF488, Thermo Fisher) in 1 mL of ethanol overnight at 4 °C. After centrifugation to remove unconjugated dye, 500 μg of CeO_2_-AF488 was mixed with 50 μg of CCM in 500 μL of DPBS and then the sample was ultrasonicated on ice for 90 s (15 s on, 15 s off, 120 W, 50% amplitude, Fisher Scientific, Model 120). The CCM-CeO_2_-AF488 nanoparticles were collected by centrifugation and washed three times with DPBS. The prepared samples were stored at 4 °C in the dark for further use.

Five ×10^5^ U-87 cells and HBMECs were seeded in 35 mm Petri dishes and incubated for 24 h. Then 10 μg/mL CeO_2_-AF488 and CCM-CeO_2_-AF488 were incubated with U-87 cells and HBMECs for 4 h, respectively. Subsequently, cells were washed with DPBS three times and detached with 0.05% EDTA–trypsin. Detached cells were collected, centrifuged, washed with DPBS three times, and immediately analyzed by a flow cytometer (Luminex Amnis Image Stream). The fluorescence intensity of the AF488 dye was used to indicate internalized nanoparticles in both HBMECs and U-87 cells.

### Subcellular Distribution

Five thousand U-87 cells were seeded into 96-well plates (Corning 3603) and incubated at 37 °C overnight. Then, 100 μg/mL AF488-labeled CCM-CeO_2_ was added and incubated for 4 h. After washing three times with DPBS, cells were stained by DAPI (10 μg/mL) and Lysobrite Red DND-99 dye (5 μg/mL, AAT Bioquest) and incubated at 37 °C for 30 min. Immediately, cells were fixed with 4% paraformaldehyde and imaged by laser scanning confocal microscopy (LSCM, ImageXpres, Molecular Devices). The Pearson’s coefficient was quantified using the JACoP plugin from ImageJ software (NIH).

### Spheroid Penetration Assay

First, 100 μg/mL CCM-CeO_2_-AF488 was added to U-87 spheroids and incubated at 37 °C overnight. After washing three times with DPBS, spheroids were fixed by 4% paraformaldehyde (Thermo Fisher Scientific) and stained by DAPI (10 μg/mL) overnight at 4 °C. The penetration of CCM-CeO_2_ into spheroids was analyzed by LSCM.

### Hypoxia and NO Staining in Spheroids

U-87 spheroids were incubated with 100 μL of 100 μg/mL CCM-CeO_2_ overnight at 37 °C and then washed by DPBS three times to remove unpenetrated nanoparticles. Hypoxia and NO were stained with 5 μM Image-iT Green hypoxia reagent and 5 μM DAR-1 for 1 h in 100 μL of DPBS at 37 °C, respectively. The fluorescent images were taken by LSCM, and the fluorescent intensity was analyzed using ImageJ (NIH).

### Immunofluorescent Staining of HIF-1α

U-87 spheroids were treated with 100 μg/mL CCM-CeO_2_ overnight at 37 °C and washed three times with DPBS. Spheroids were fixed and permeabilized with 4% paraformaldehyde and 0.5% Triton X-100 (Thermo Fisher Scientific) at room temperature for 1 h, respectively. Then spheroids were washed by DPBS and blocked by 1% BSA overnight at 4 °C. Finally, the spheroids were stained with DAPI (10 μg/mL) and Alexa Fluor 647-conjugated mouse antihuman HIF-1α (5 μg/mL, BD Pharmingen). The spheroids were imaged by LSCM, and the fluorescence intensity of HIF-1α was quantified by ImageJ.

### In Vitro Drug Release Study

First, 100 μg/mL CCM-CeO_2_-TMZ was resuspended in DPBS, placed in a D-Tube Dialyzer Midi instrument with a cutoff molecular weight 3 kDa (Sigma-Aldrich), and gently shaken at 37 °C. Drug release at pH 5 and 7.4, with and without lysosomal protease cathepsin B, was explored. Released TMZ was collected at different time points and determined by a Nanodrop One instrument (Thermo Fisher Scientific).

### Cellular Oxidative Stress Staining

U-87 cell spheroids were incubated with 200 μg/mL CCM-CeO_2_, CCM-CeO_2_-TMZ, and an equivalent amount of TMZ for 3 days. After being washed three times by DPBS, cell spheroids were stained with CellROX Deep Red reagent (5 μM) and DAPI (10 μg/mL) for 1 h at room temperature. Immediately after staining, spheroids were imaged by confocal microscopy and fluorescence intensity was quantified using ImageJ.

### Lysosome Membrane Permeabilization Assay

Cell spheroids were formed by culturing 10,000 cells in an ultralow attachment 96-well plate for 3 days and treated with 200 μg/mL CCM-CeO_2_, CCM-CeO_2_-TMZ, and an equivalent amount of TMZ for 3 days. After being washed three times with DPBS, cell spheroids were stained with DAPI (10 μg/mL) and acridine orange solution (10 μg/mL) and incubated at room temperature for 1 h. Cell spheroids were analyzed by confocal microscopy. The lysosomes with integrated membranes were quantified by fluorescence intensity using ImageJ.

### Apoptosis/Necrosis Assay

U-87 cell spheroids were first treated with 400 μg/mL CCM-CeO_2_, CCM-CeO_2_-TMZ, and an equivalent amount of TMZ for 48 h. Subsequently, cell spheroids were washed with DPBS and dissociated with an Accutase/EDTA–trypsin mixture (Sigma-Aldrich) at 37 °C until most cells were dissociated from spheroids. The dissociated cells were centrifuged and washed with DPBS and stained by a Cell Meter Apoptotic and Necrotic Multiplexing Detection Kit (AAT Bioquest) at room temperature for 30 min. After DPBS washing, a flow cytometer was used to analyze cells (Luminex Amnis Image Stream).

### In Vitro Antitumor Efficacy Assay

To evaluate the antitumor efficacy of TMZ and CCM-CeO_2_-TMZ in vitro, U-87 spheroids were treated with various concentrations of CCM-CeO_2_, TMZ, and CCM-CeO_2_-TMZ for 10 days. The drug solutions were changed every 2 days, and after 10 days, cell spheroids were washed three times by DPBS. Then, 1× Calcein AM was prepared per the manufacturer’s protocol and added to spheroids. After incubation at room temperature for 1 h, spheroids were washed with DPBS, and the fluorescence intensity of Calcein AM was measured by a microplate reader (excitation wavelength, 488 nm; emission wavelength, 515 nm). The viability of the treated spheroids was normalized to that of untreated spheroids. The IC_50_ values of free TMZ and CCM-CeO_2_-TMZ were calculated using Prism GraphPad 7.0.

### Statistical Analysis

All data were presented as the mean ± standard deviation. The statistical significance between two groups was analyzed by Student’s *t*-test, and statistical difference among multiple groups was determined by one-way ANOVA using GraphPad Prism 7.0. A *P*-value of <0.05 was considered statistically significant.

## Results and Discussion

### Synthesis and Characterizations of CeO_2_ and CCM-CeO_2_

**Figure 1:**
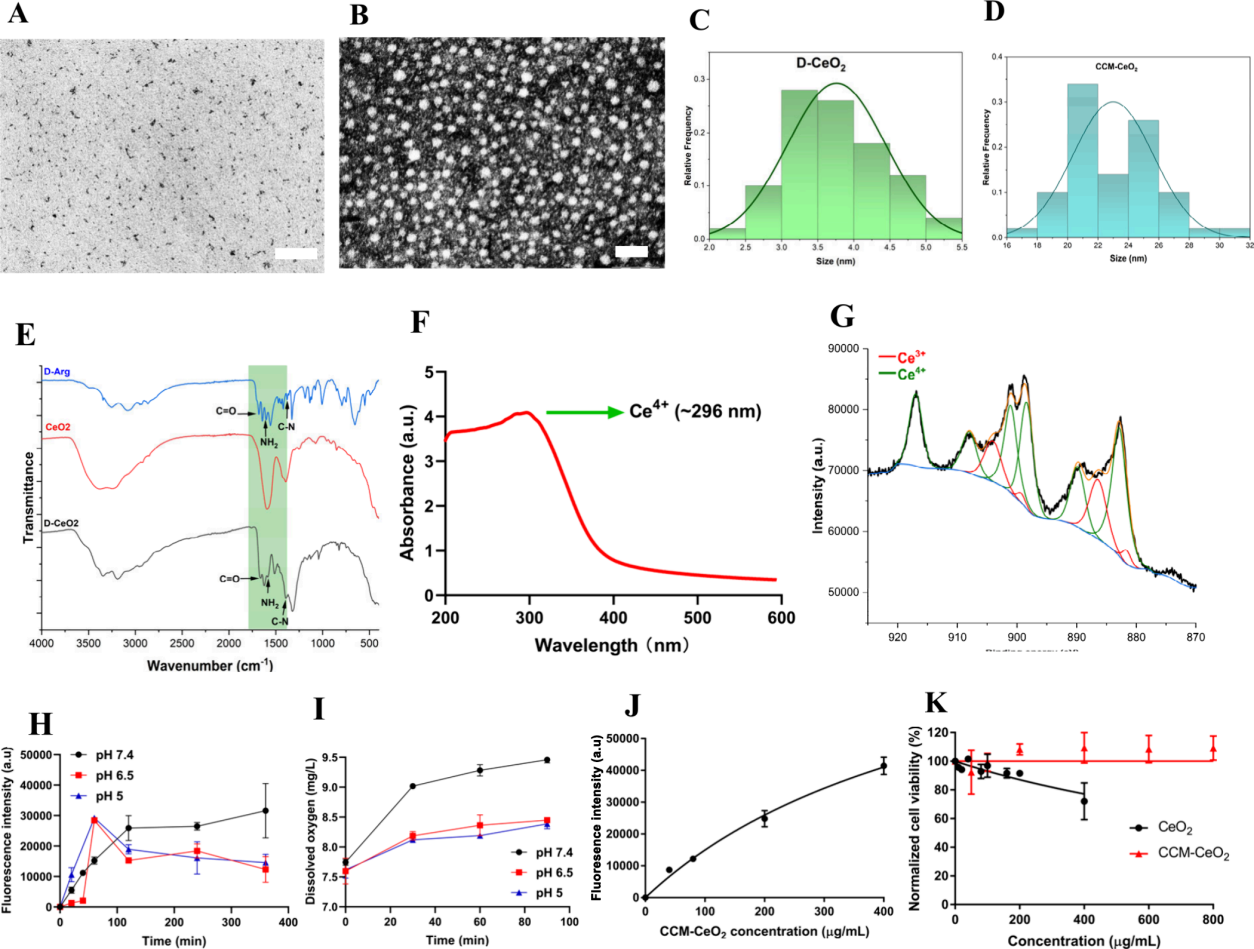
Characterization of d-CeO_2_ and CCM-CeO_2_. (A and B) TEM images of CeO_2_ and negatively stained CCM-CeO_2_. (C and D) Size distribution of d-CeO_2_ and CCM-CeO_2_. (E) FTIR analysis of d-arginine, synthesized d-CeO_2_, and CeO_2_ control obtained from Alfa Aesar. (F) UV–vis scanning of d-CeO_2_. (G) XPS analysis of d-CeO_2_. (H) NO produced by reaction between CCM-CeO_2_ and H_2_O_2_ at different pH values. (I) Dissolved oxygen produced by decomposition of H_2_O_2_ in DPBS at different pH values. (J) Total consumed H_2_O_2_ by CCM-CeO_2_. (K) Cell viability assays of CeO_2_ and CCM-CeO_2_ using cancer spheroids. Scale bar: 50 nm.

Nanoceria is a special type of nanoparticle that can mimic the activities of several natural enzymes, including SOD, catalase, peroxidase, oxidase, etc. The antioxidant activity of nanoceria involves cycling between Ce^3+^ and Ce^4+^ ions on the nanoceria surface. Nanoceria with a low ratio of Ce^4+^/Ce^3+^ is more SOD-mimetic, while nanoceria with a high ratio of Ce^4+^/Ce^3+^ is catalase-favorable. Moreover, nanoceria synthesized by alkaline precipitation can increase the Ce^4+^/Ce^3+^ ratio, which further increases the catalase activity.^52^ Instead of using harsh alkaline solutions, here we used the basic amino acid d-arginine to synthesize nanoceria. d-Arginine can also serve as a capping agent to ensure a good dispersion of nanoceria. The TEM images showed that the bare CeO_2_ was well dispersed with a diameter of ∼4 nm and after CCM coating, the diameter increased to ∼22 nm (Figures 1A–1D). Additionally, atomic force microscopy (AFM) was used to characterize the morphology and size of CCM-CeO_2_. As shown in Figure S2, CCM-CeO_2_ was spheric, and its diameter was ∼14.2 nm, slightly smaller than the size analyzed by TEM. FTIR analysis showed that d-arginine-modified CeO_2_ (d-CeO_2_) displayed some characteristic peaks of d-arginine (Figure 1E). The peak at ∼1660 cm^–1^ corresponds to the C=O stretching vibration of the carboxyl group, and the band at 1396 cm^–1^ is attributed to the stretching vibration of C–N. The peak between 1680 and 1600 cm^–1^ belongs to the NH_2_-bending vibration.^53^ Overall, these peaks confirmed the d-arginine coating on the CeO_2_ surface.

Next, we investigated Ce^3+^ and Ce^4+^ on the surface of d-CeO_2_ by UV–vis scanning. Ce^3+^ and Ce^4+^ have absorbance peaks at ∼250 and ∼296 nm, respectively.^54^ As shown in Figure 1F, only an absorbance peak at ∼296 nm was observed, indicating a high concentration of Ce^4+^ on the surface of d-CeO_2_. To further quantify the Ce^4+^/Ce^3+^ ratio, X-ray photoelectron spectroscopy (XPS) was performed. The full survey and fine scans of C and N elements (Figure S1) verified the existence of d-arginine on the surface of d-CeO_2_, which is consistent with our FTIR results. In Figure 1G, the XPS analysis revealed the coexistence of Ce^3+^ and Ce^4+^ ions on the d-CeO_2_ surface. As summarized in Table S1, the peaks at 881.6, 886.5, 899.3, and 904 eV are assigned to Ce^3+^, while peaks at 882.9, 889.9, 898.4, 901.1, 908, and 917 eV are assigned to Ce^4+^. The estimated ratio of Ce^4+^/Ce^3+^ was 3.69, indicating a high catalase activity of d-CeO_2_.

Unlike l-arginine, d-arginine is metabolically inert without interfering with other cellular signaling pathways, thus avoiding possible side effects of l-arginine.^55^ Although d-arginine cannot be metabolized by tumor cells, it can nonenzymatically react with H_2_O_2_ in the TME to produce nitric oxide (NO).^56^ The NO production from the reaction between d-arginine on the surface of CeO_2_ and 100 μM H_2_O_2_ was detected by DAR-1. At an acidic pH, nanoceria exhibited low catalase activity and consumed less H_2_O_2_, which led to more NO production. After 1 h, NO concentration was decreased at acidic pH due to the fact that NO tends to escape in an acidic solution (Figure 1H).^57^ The dissolved oxygen level was increased after the decomposition of H_2_O_2_ by CeO_2_ even with CCM coating, increasing from 7.75 ± 0.05 to 9.46 ± 0.05 mg/L at neutral pH. While acidic pH impaired the oxygen-generating capacity of nanoceria because of inhibited catalase activity, only a slight increase of dissolved oxygen was observed for pH 6.5 and 5 (Figure 1I). Then the consumed H_2_O_2_ by 100 μg/mL CCM-CeO_2_ was also evaluated by a hydrogen peroxide assay kit and the total consumption of H_2_O_2_ was increased with CCM-CeO_2_ concentration increasing (Figure 1J). The results showed that after CCM coating, d-arginine-modified CeO_2_ still retained its catalase-like and nonenzymatic activities to produce O_2_ and NO, and the gas production could be triggered by H_2_O_2_.

To further evaluate the biocompatibility of synthesized d-CeO_2_ and CCM-CeO_2_, a cytotoxicity assay was performed by using 3D cell spheroids. Cell spheroids with a large diameter (>400 μm) display layer-like structure, with necrotic cells in the core, analogue to the poorly vascularized regions of solid tumors. Surrounding the necrotic core, there is a layer of quiescent cells and an outside layer of highly proliferating cells.^58,59^ Therefore, large-diameter spheroids can create oxygen, nutrition, and other physiochemical gradients, similar to those in solid tumors, and can be employed for in vitro cancer research.^60^ As shown in Figure 1K, for the as-synthesized d-CeO_2_, more than 91% of cells were viable at concentrations <200 μg/mL. However, at 400 μg/mL, the viability of U-87 cells decreased to 72%. In contrast, CCM-CeO_2_ did not exhibit any cytotoxicity even at 800 μg/mL. Overall, these results demonstrated that CeO_2_ could be facilely synthesized using d-arginine and that CCM-CeO_2_ still retained its activities toward H_2_O_2_. More importantly, CCM camouflaging notably improved the biocompatibility of CeO_2_ which makes CCM-CeO_2_ a good drug carrier for cancer therapy.

### SOD, Oxidase, and Peroxidase Activities of CCM-CeO_2_

**Figure 2:**
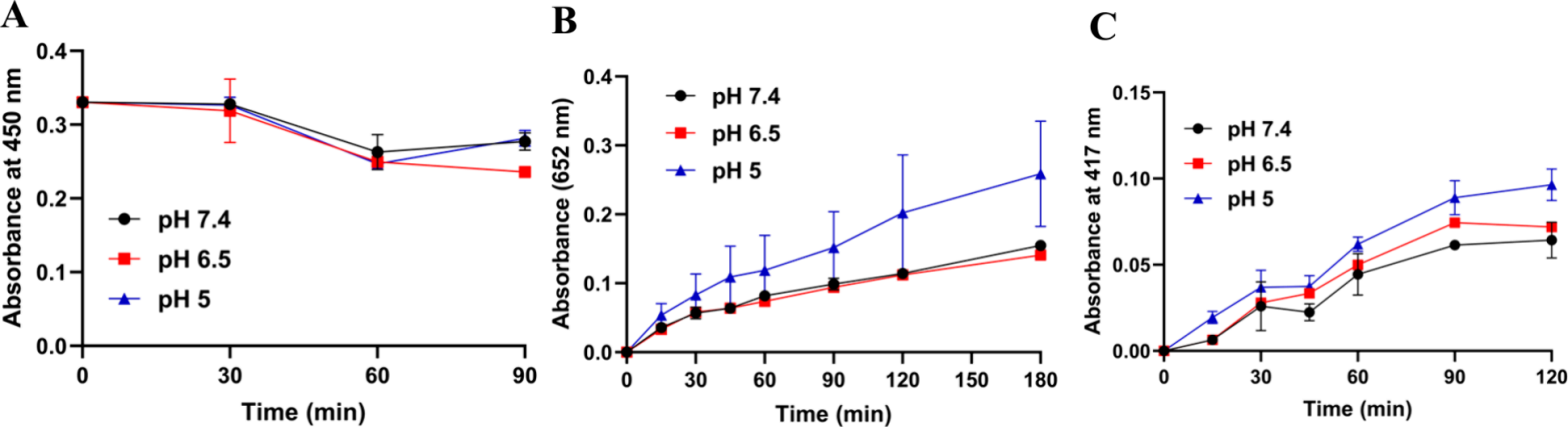
Time-dependent enzymatic assays of CCM-CeO_2_ at different pHs. (A) SOD activity assay using a SOD assay kit. (B) Oxidase activity assay using TMB. (C) Peroxidase assay was performed using an OPD.

Nanoceria with a high Ce^4+^/Ce^3+^ ratio exhibits high catalase activity and low SOD activity. For the SOD assay, high SOD activity reduced the production of yellow products, which resulted in decreased absorbance at 450 nm. The as-synthesized CCM-CeO_2_ barely reduced the absorbance at 450 nm within 90 min, which indicated a low SOD activity. Moreover, the pH had little effect on the SOD activity (Figure 2A). For oxidase and peroxidase, the produced ROS could convert colorless dyes into their oxidized states, displaying a blue color for oxidized TMB and a yellow color for oxidized OPD, respectively. As shown in Figure 2B, only mildly acidic pH (pH 5) increased the oxidase activity, as indicated by the increased absorbance of oxidized TMB. For peroxidase activity, although the absorbance increased with decreasing pH, the absorbance change was minimal. Considering the hypoxic tumor microenvironment (TME), d-CeO_2_ exhibits a low pro-oxidant property.

### Homotypic Targeting and Cellular Internalization of CCM-CeO_2_

**Figure 3:**
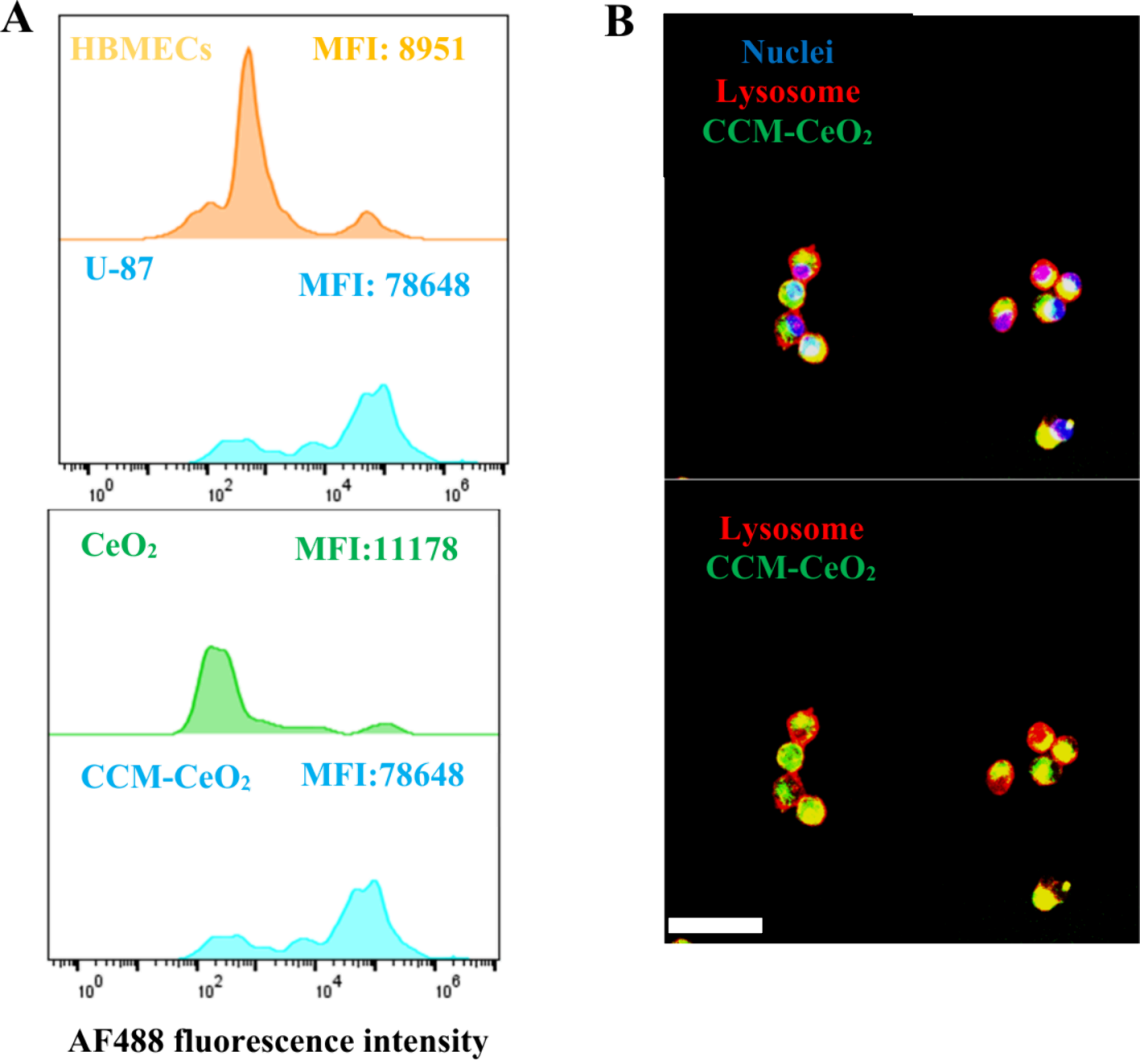
Homotypic targeting and subcellular distribution of CCM-CeO_2_. (A) Flow cytometric analysis of the cellular uptake of CeO_2_-AF488 and CCM-CeO_2_-AF488. The uptake of CCM-CeO2-AF488 in HBMECs and U-87 cells (up). The uptake of CeO_2_-AF488 and CCM-CeO_2_-AF488 in U-87 cells (bottom). (B) Subcellular distribution of CCM-CeO_2_. U-87 cells were treated with 10 μg/mL AF488-labeled CCM-CeO_2_ for 4 h and then stained by LysoBrite Red DND-99. Blue fluorescence indicates nucleus; green fluorescence indicates CCM-CeO_2_; red fluorescence indicates lysosome; scale bar, 100 μm.

Specifically, targeting cancer cells not only reduces systematic toxicity but also improves the therapeutic efficacy. One common strategy for targeting cancer cells is to modify drug carriers with targeting ligands, such as heavy-chain ferritin^61−63^ and folate,^64^ to specifically interact with receptors overexpressed on the cancer cells. Although effective, this method requires a complex chemical conjugation process. Here, we utilized a facile strategy using cancer cell membranes to target GBM cells specifically. To demonstrate that GBM cell-membrane-camouflaged CeO_2_ could effectively target GBM cells, the cellular uptake of 10 μg/mL AF488 dye-labeled CCM-CeO_2_ in U-87 cells and human brain microvascular endothelial cells (HBMECs) for 4 h was compared by a flow cytometer. The targeting efficiency of CCM-CeO_2_ toward U-87 cells was approximately 8 times higher than that toward HBMECs (mean AF488 fluorescence intensity 78,648 for U-87 cells vs 8951 for HBMECs) (Figure 3A). The high targeting efficiency is due to the multiple ligands on CCM, and thus CCM has higher targeting efficiency than a single ligand-based strategy.^65^ To further demonstrate that CCM coating can improve the tumor targetability, U-87 cellular uptake of coated and uncoated CeO_2_-AF488 nanoparticles was also investigated. CCM-CeO_2_ nanoparticles exhibited ∼6 times higher targeting efficiency compared to uncoated CeO_2_ nanoparticles (mean AF488 fluorescence intensity 78,648 for CCM-CeO_2_ vs 11,178 for CeO_2_.

Cellular internalization efficiency and subcellular distribution of drugs/drug carriers also determine the therapeutic efficacy.^66^ To investigate the subcellular distribution of CCM-CeO_2_, AF488-labeled CCM-CeO_2_ were incubated with U-87 cells, and nuclei and lysosomes were stained. The colocalization of CCM-CeO_2_ (red fluorescence) and lysosomes (green) was observed under confocal microscopy with a quantified Pearson’s coefficient of ∼0.80 (Figure 3B) analyzed by the JACoP plugin in ImageJ, implying that most internalized CCM-CeO_2_ nanoparticles were localized in lysosomes. While TMZ represents the first-line chemotherapy for GBM, it suffers from aqueous instability. TMZ is rapidly hydrolyzed to 5-(3-methyltriazen-1-yl) imidazole-4-carboxamide (MTIC) at physiological pH but stable at acidic pH (pH < 5).^67^ The lysosomal distribution of the CCM-CeO_2_ carrier facilitates the delivery of TMZ to lysosomes, which favors TMZ therapy due to the unique acidic stability of TMZ.

### Penetration of CCM-CeO_2_ in Spheroids

**Figure 4:**
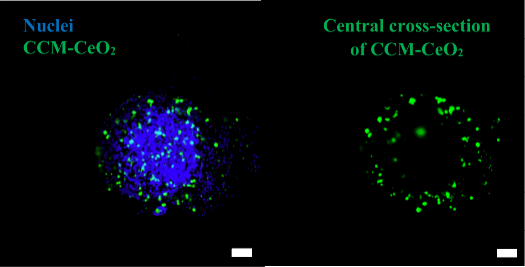
Penetration of CCM-CeO_2_ in GBM spheroids. Left: Z-stack fluorescent image of penetrated CCM-CeO_2_ in U-87 spheroids. Green fluorescence indicates CCM-CeO_2_, and blue fluorescence indicates nucleus. Right: Central cross-section of CCM-CeO_2_ penetrated spheroid. Scale bar: 100 μm.

Penetration of drug/drug carriers is one of the most crucial factors that determine the overall therapeutic efficacy in solid tumors.^68,69^ In various solid tumor models, deeper penetration of the chemotherapeutic drug doxorubicin (DOX) correlates with better antitumor efficacy and the characteristic penetration depth (the distance from the nearest vessel where the DOX fluorescence intensity decreases to 50%) is ∼40–50 μm.^70^ To further increase the penetration depth of chemotherapeutic agents, loading drugs into nanocarriers might be a promising strategy. As shown in Figure 4, the AF488-labeled CCM-CeO_2_ penetrated U-87 spheroids with a distance of 148.3 ± 31 μm. It is worth noting that the most significant distance that loaded TMZ could penetrate is likely greater than 148 μm, considering that TMZ was released there and diffused into the spheroid core.

### Alleviation of Hypoxia

**Figure 5:**
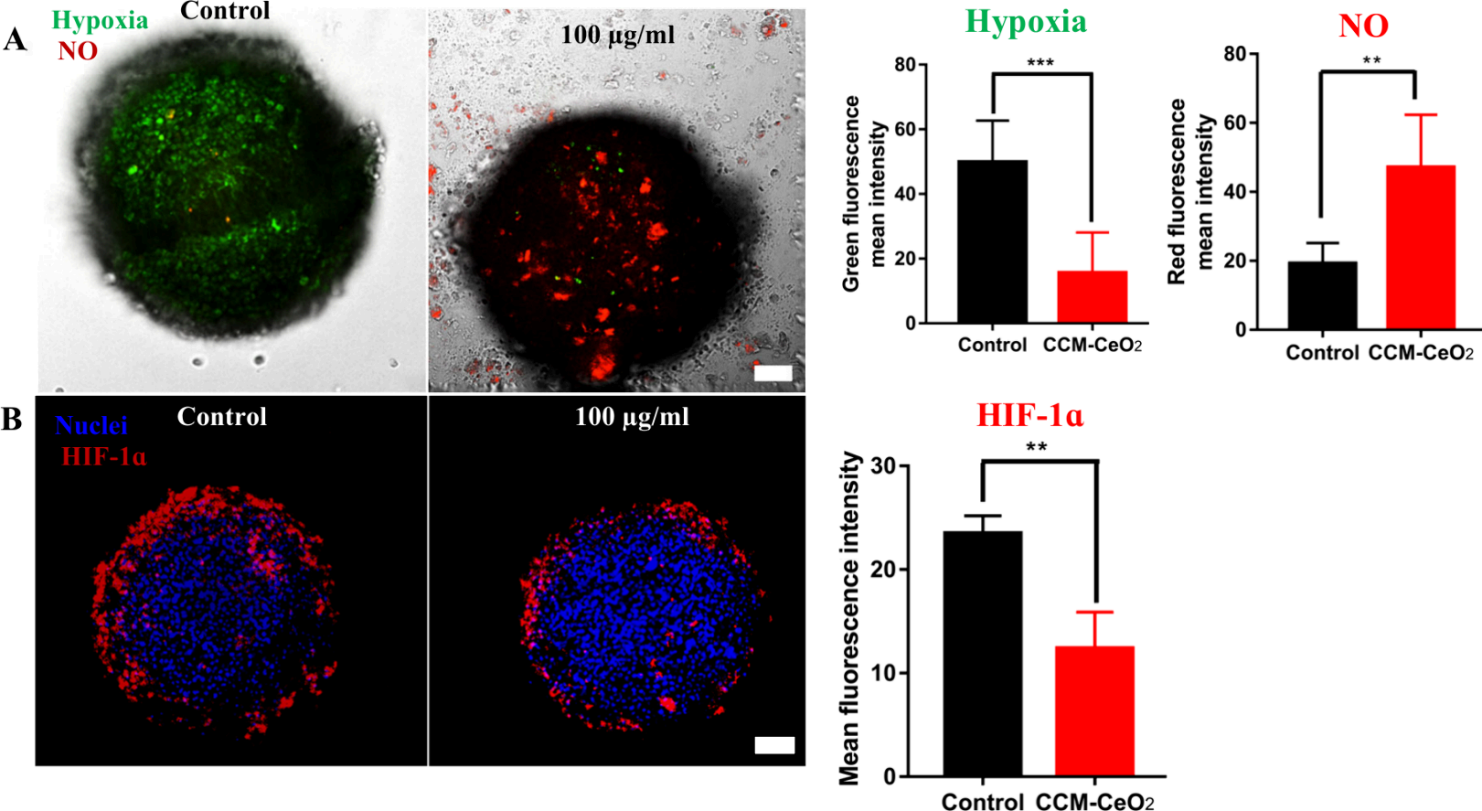
Hypoxia alleviation in spheroids. (A) Hypoxia and NO stained by Image-iT hypoxia reagent (green) and DAR-1 (red), respectively. (B) Immunofluorescent staining of HIF-1α protein. All fluorescence intensities were quantified by ImageJ. **p < 0.01, ***p < 0.001; scale bar: 100 μm.

It is known that CCM-CeO_2_ could produce NO and O_2_ with the presence of 100 μM H_2_O_2_ (Figures 1 H and 1I). After examining the penetration of CCM-CeO_2_, whether the penetrated CCM-CeO_2_ could generate O_2_ and NO in spheroids was studied here, given that large spheroids can mimic the H_2_O_2_ gradient. The Image-iT hypoxia reagent displays green fluorescence when the O_2_ level is <5%, and its fluorescence intensity decreases as the O_2_ level increases. For NO staining, the fluorescence intensity of DAR-1 increases as the NO level increases. The control spheroids showed a higher hypoxia fluorescence intensity, indicating that spheroids with a large diameter are feasible to mimic the hypoxic solid tumors. After 100 μg/mL CCM-CeO_2_ treatment, hypoxia was relieved and green fluorescence intensity significantly decreased. The NO level was also significantly increased, implying the generation of aqueous O_2_ and NO in spheroids (Figure 5A). The results indicated the TME-responsive generation of aqueous O_2_ and NO in spheroids.

To explore whether the produced O_2_ and NO resulted in the decreased expression of the HIF-1α protein, an immunofluorescent staining assay was performed in U-87 spheroids. Control spheroids had significantly higher expression of HIF-1α than 100 μg/mL CCM-CeO_2_-treated spheroids (Figure 5B), indicating that our nanoparticles can effectively alleviate hypoxia in spheroids. Consistent with a previous study,^71^ the red fluorescence of HIF-1α was mainly seen at the periphery ring of the spheroids where peri-necrotic, proliferating cells have resided, which might be contrary to the fact that the inner core of spheroids is more severely hypoxic. Considering that the O_2_ levels of periphery tissue range from 3.4% to 6.8%, depending on the tissue type,^72^ the outside layer of proliferating cells may also express HIF-1α in U-87 spheroids. In addition, different from the small-molecule hypoxia detection reagent (Figure 5A), the mouse antihuman HIF-1α protein has a relatively large molecular weight, and its diffusion into the inner core of spheroids is notably limited, which might also explain that HIF-1α was mainly seen in the peripheral spheroids. In contrast, the HIF-1α in a cryostat-sectioned GBM spheroid^73^ and another cancer spheroid^74^ was mainly located in the center of the thin section.

Previous studies on nanoceria-enhanced chemotherapy attributed the efficacy to the oxidase-like activity of nanoceria and the resultant increased ROS production.^29−31^ Since the oxidase-catalyzed reaction is an O_2_-consuming process, it aggravates hypoxia. However, in this study, alleviated hypoxia was observed in spheroids (Figure 5), which likely indicates that our nanoceria are more favorable for catalase activity than oxidase activity.

### Drug Loading and Drug Release Profiles of CCM-CeO_2_-TMZ

**Figure 6:**
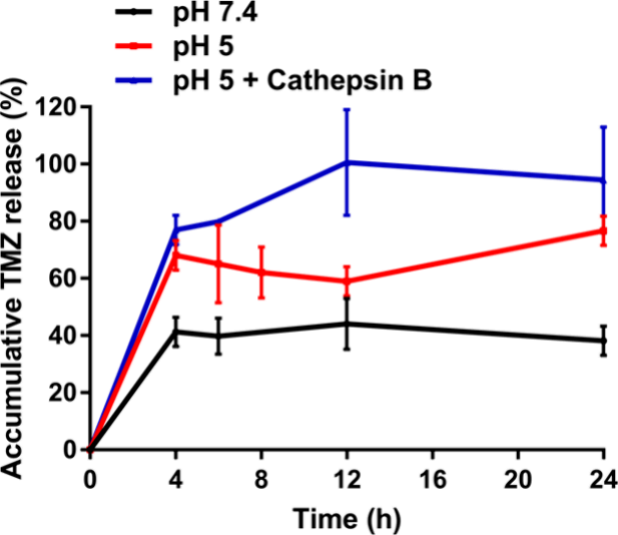
In vitro TMZ release under different conditions.

After exploration of the subcellular distribution and hypoxia-alleviating activity of CCM-CeO_2_, the performance of CCM-CeO_2_ as a drug carrier was evaluated. TMZ was used as the model drug, and the drug loading efficiency and drug loading capacity of CCM-CeO_2_ were 21.3 ± 4.1% and 29.9 ± 7.5%, respectively. Since most CCM-CeO_2_ accumulated in lysosomes, the effects of acidic pH (5) and lysosomal protease (cathepsin B) on TMZ release profiles were also examined. As shown in Figure 6, the accumulative release profile of TMZ was first studied at pH 7.4, which showed that only ∼40% of TMZ was released within 24 h. At acidic pH 5, the release was accelerated, and ∼80% TMZ was released within 24 h. Lysosomal protease cathepsin B also accelerated the release of TMZ, and 94% of TMZ was released within 24 h. These results indicated that the lysosomal environment (acidic pH and lysosomal protease) triggered a rapid TMZ release.

### Cellular Oxidative Stress and LMP Induced by CCM-CeO_2_

**Figure 7:**
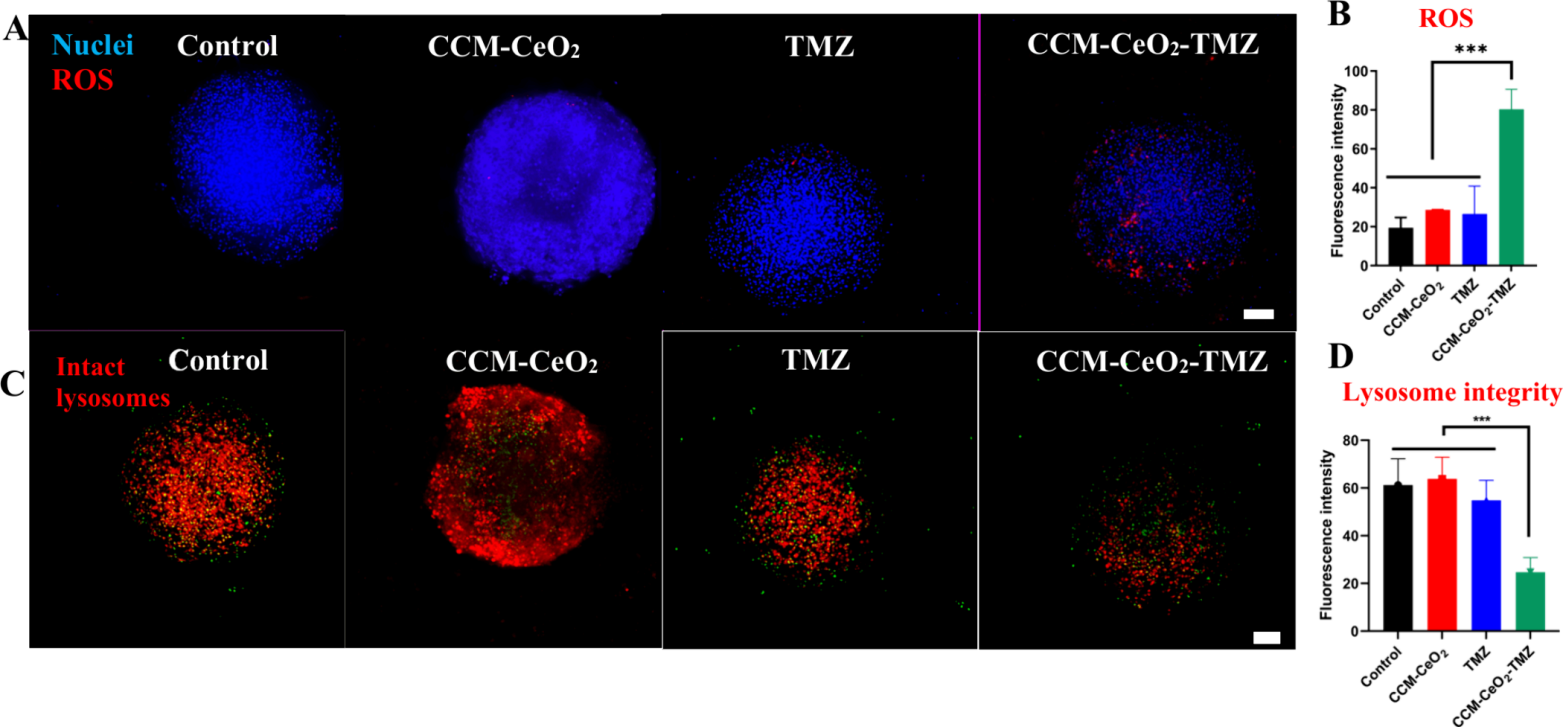
Oxidative stress staining and the lysosome permeabilization assay. (A) Oxidative stress staining by CellROX Deep Red reagent. Blue fluorescence indicates nuclei and red fluorescence indicates ROS. (B) Quantification of ROS fluorescence intensity by ImageJ. (C) OA staining. Red fluorescence indicates intact lysosomes. (D) Quantification of lysosome integrity by ImageJ. ***p < 0.001; scale bar: 100 μm.

Intracellular ROS has been associated with chemoresistance, and the acquisition of chemoresistance in gliomas is linked to an increased level of mitochondrial coupling and decreased level of ROS production. Previous studies showed that TMZ caused DNA damage and led to ROS production.^75^ Furthermore, ROS induced by TMZ is more pronounced in TMZ-sensitive glioma cells, rather than in TMZ-resistant glioma cells.^76,77^ On the other hand, mitochondrial ROS scavenger abrogated TMZ efficacy in TMZ-sensitive glioma cells. These studies demonstrate the therapeutic potential of increasing the ROS production to overcome GBM chemoresistance. To determine if TMZ could induce ROS and CCM-CeO_2_ could amplify the ROS generation of TMZ, we performed the oxidative stress staining using CellROX Deep Red reagent in spheroids. As shown in Figure 7A, more ROS was induced after CCM-CeO_2_-TMZ treatment compared to TMZ and CCM-CeO_2_ treatment, and the elevated ROS is likely due to the alleviation of hypoxia by CCM-CeO_2_.

Since CCM-CeO_2_ is preferentially localized in lysosomes, which are more susceptible to ROS stimulation, the effects of increased ROS on lysosome membrane integrity were evaluated. The lysosome membrane permeabilization (LMP) assay was performed using acridine orange (AO) staining. AO is a lysosomotropic dye that emits red fluorescence at high concentrations when accumulated in lysosomes. Upon stimulation that damages lysosome membranes, AO dissipates throughout the cytosol and emits green fluorescence at lower concentrations.^51^ Consequently, the red fluorescence of AO indicates intact lysosomes, while the green fluorescence indicates damaged lysosomes. Although the green fluorescence of AO staining denotes the damaged lysosomes, stimulation does not necessarily lead to the increased green fluorescence in cytosol.^78,79^ It is likely that once the LMP is provoked, acidic contents and lysosomal protease could damage the cell membrane, bringing about the release of AO stain into the extracellular space. As shown in Figure 7C, AO stain in green fluorescence was diffused out of spheroids in TMZ- and CCM-CeO_2_-TMZ-treated groups. Therefore, this study used the decrease in red fluorescence as an indicator for LMP. TMZ treatment slightly decreased the red fluorescence without statistical significance while CCM-CeO_2_-TMZ induced a significant drop in red fluorescence. CCM-CeO_2_ and TMZ alone preserved the lysosome membrane integrity (Figure 7C), which was consistent with a previous report. In that study, TMZ alone only increased lysosome volume, and lysosomal enzyme cathepsin B was retained in lysosomes, not inducing LMP.^80^

### Apoptosis/Necrosis Assay and In Vitro Antitumor Efficacy Assay

**Figure 8:**
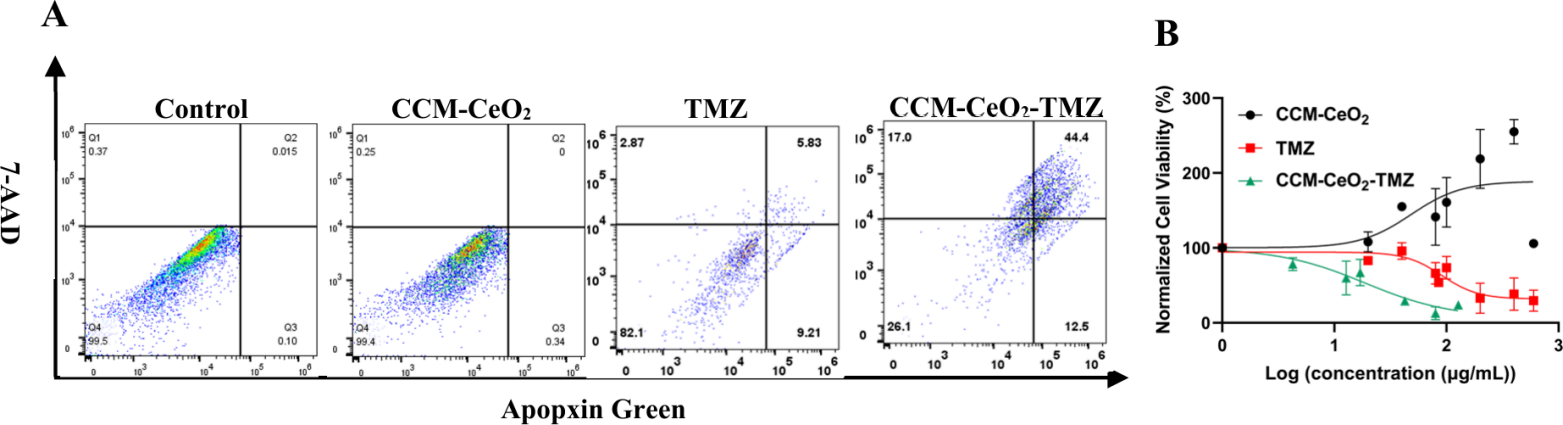
Apoptosis/necrosis assay and in vitro antitumor efficacy assay. (A) Apoptosis/necrosis ratios of cells under different conditions were analyzed by flow cytometry. Apoptotic cells were stained by Apopxin Green and necrotic cells were stained by 7-AAD. (B) In vitro antitumor efficacy assay. Cell viability of free TMZ, CCM-CeO_2_, and CCM-CeO_2_-TMZ was determined by the fluorescence intensity of Calcein AM.

After we showed that CCM-CeO_2_ was a biocompatible drug carrier without triggering ROS production or inducing LMP, we next explored its therapeutic effects as a drug carrier. Depending on its severity, LMP is an important process that can induce cell apoptosis or necrosis. Moderate LMP induces apoptosis, while extensive LMP triggers necrosis.^81^ To investigate the effects of ROS induced by CCM-CeO_2_-TMZ, we performed an apoptosis/necrosis assay. During early apoptosis, phosphatidylserine (PS) is translocated from the inner leaflet to the outer leaflet of the plasma membrane. Annexin V can efficiently and specifically bind to PS via a Ca^2+^-dependent manner and thus can be used for quantifying early apoptotic cells.^82^ 7-AAD is a nonvital DNA dye and can discriminate late apoptotic cells and necrotic cells which lose membrane integrity.^83^ In this assay, GBM spheroids were first treated by CCM-CeO_2_, TMZ, and CCM-CeO_2_-TMZ and then dissociated by the Accutase/EDTA–trypsin mixture. The dissociated cells were stained by a Cell Meter Apoptotic and Necrotic Multiplexing Detection Kit and analyzed by flow cytometry. In Figure 8A, the ratio of apoptosis/necrosis cells for TMZ was ∼17.91% (∼15.04% apoptosis and 2.87% necrosis), while for CCM-CeO_2_-TMZ treated cells, the ratio of apoptosis/necrosis was 73.9% (56.9% apoptosis and 17% necrosis). For the control and CCM-CeO_2_ groups, no noticeable apoptosis or necrosis was observed.

These results are consistent with other findings that TMZ alone can only induce apoptosis of a small cell population and the predominant effect of TMZ on U-87 cells is G_2_-M cell arrest and autophagy, not apoptosis.^80,84^ When exposed to TMZ, the fate of GBM cells is determined by the p53 status. U-87 cells have wild-type p53 and thus TMZ only induces prolonged G_2_-M cell arrest and growth arrest in U-87 cells, which leaves most cells viable yet nonproliferative.^84^ After CCM-CeO_2_-TMZ treatment, we observed increased apoptosis and necrosis in U-87 cells, implying that CCM-CeO_2_ facilitated apoptosis and necrosis in the TMZ-treated U-87 cells. The apoptosis and necrosis promoted by CCM-CeO_2_ may be attributed to increased ROS production within U-87 cells as a body of literature has shown that oxidative stress could lead to apoptosis and necrosis by lysosomal rupture.^85−87^

It is well established that hypoxia contributes to chemoresistance and common chemotherapeutics produce less ROS under hypoxia, while normoxia or alleviation of hypoxia can increase the ROS produced by chemotherapeutics.^39,77^ Interestingly, CCM-CeO_2_ was found to promote the cell proliferation of U-87 spheroids, likely attributed to the cell-stimulating effects of oxygen and low NO levels.^88^ We evaluated the antitumor efficacy of CCM-CeO_2_-TMZ in vitro using spheroids and compared it to free TMZ. After 10 days of treatment, the IC_50_ values of free TMZ and CCM-CeO_2_-TMZ were 174.5 and 42.6 μg/mL, respectively (Figure 8B). The superior antitumor efficacy of CCM-CeO_2_-TMZ over TMZ may be attributed to increased spheroid penetration, targeted lysosomal delivery, increased ROS production, etc.

Oxygenation or hyperbaric oxygen has been well documented to enhance chemotherapy, while the effects of NO on therapeutic efficacy are still obscure. A previous study has revealed that endogenous NO signaling was involved in hypoxia-induced drug resistance and reactivation of the NO signaling pathway attenuated drug resistance.^43^ Another study also found that a high level of NO promoted drug-induced apoptosis by inhibiting NF-κB and further downstream anti-apoptotic gene products.^41^ Besides its effects on HIF-1α, NO can synergize with TMZ to inhibit GBM cell growth by inducing apoptosis^44^ and reducing the expression of mutated p53 and MGMT.^45^ Moreover, NO donors can also potentiate chemotherapy by increasing vascular and blood–brain tumor barrier permeability^89,90^ and further enhancing the delivery of chemotherapeutics into GBM. On the other hand, peroxynitrite, a highly reactive metabolite of NO and superoxide ions (O^2–^), may contribute to the loss of wide-type p53 functional activity in glioma cells by posttranslationally modifying the wild-type p53 protein and inhibiting its DNA-binding ability at physiologically relevant concentrations.^91,92^ These modifications dysregulate the p53 protein and render GBM cells more resistant to DNA-damaging chemotherapeutics. Further transduction of wild-type p53 proteins into chemoresistant GBM cells recovered the chemosensitivity of GBM cells.^93^ Although NO can relieve hypoxia, the specific roles of NO in anti-GBM therapy require further investigation.

## Conclusions

TMZ is the standard chemotherapy for GBM, yet it has limited efficacy. Specific targeting, especially organelle targeting, and alleviating hypoxia-mediated resistance are critical to improving its bioavailability and therapeutic efficacy. The cancer-cell-membrane-coated nanoceria synthesized in this study specifically targeted GBM cells and preferentially accumulated in lysosomes. The simultaneous release of NO and O_2_ from CCM-CeO_2_ was triggered by excessive H_2_O_2_ in the tumor microenvironment, which sensitized TMZ chemotherapy by inducing intracellular ROS, damaging lysosomal membrane integrity, and further triggering apoptosis and necrosis. This study elucidated that chemotherapy enhanced by our synthesized nanoceria was more likely to be attributed to its catalase-like activity. Overall, this work provides a facile targeting and hypoxia-alleviating strategy to potentiate chemotherapy for cancer therapy.

## Supporting Information Available

Additional experimental details of material characterizations, including XPS analysis and AFM image of nanoparticles (PDF)
