# Supplementary material for: Oxygen/Nitric Oxide Dual-Releasing Nanozyme for Augmenting TMZ-Mediated Apoptosis and Necrosis
Source: Mol Pharm. 2024 Nov 21;22(1):168–80. doi: 10.1021/acs.molpharmaceut.4c00817 (PMC11707740; doi:10.1021/acs.molpharmaceut.4c00817)
Supplement: Supplementary file 1 — mp4c00817_si_001.pdf [file mp4c00817_si_001.pdf]

Supporting Information

Oxygen/Nitric Oxide Dual-Releasing Nanozyme for Augmenting TMZ-mediated Apoptosis and Necrosis

Jun Ma[1], *Jingjing Qiu*[2,3]*, *Gus A. Wright*[4], *Shiren Wang*[1,3,5]*

1. Department of Biomedical Engineering, Texas A&M University, College Station, TX 77843, United States
2. Department of Mechanical Engineering, Texas A&M University, College Station, TX 77843, United States
3. Department of Materials Science and Engineering, Texas A&M University, College Station, TX 77843, United States
4. Flow Cytometry Facility, School of Veterinary Medicine and Biomedical Sciences, Texas A&M University, College Station, TX 77843, United States
5. Department of Industrial and Systems Engineering, Texas A&M University, College Station, TX 77843, United States

* Corresponding authors:
Jingjing Qiu Tel: 979-458-6290 E-mail: jennyqiu@tamu.edu

Shiren Wang Tel: 979-458-2357 E-mail: s.wang@tamu.edu

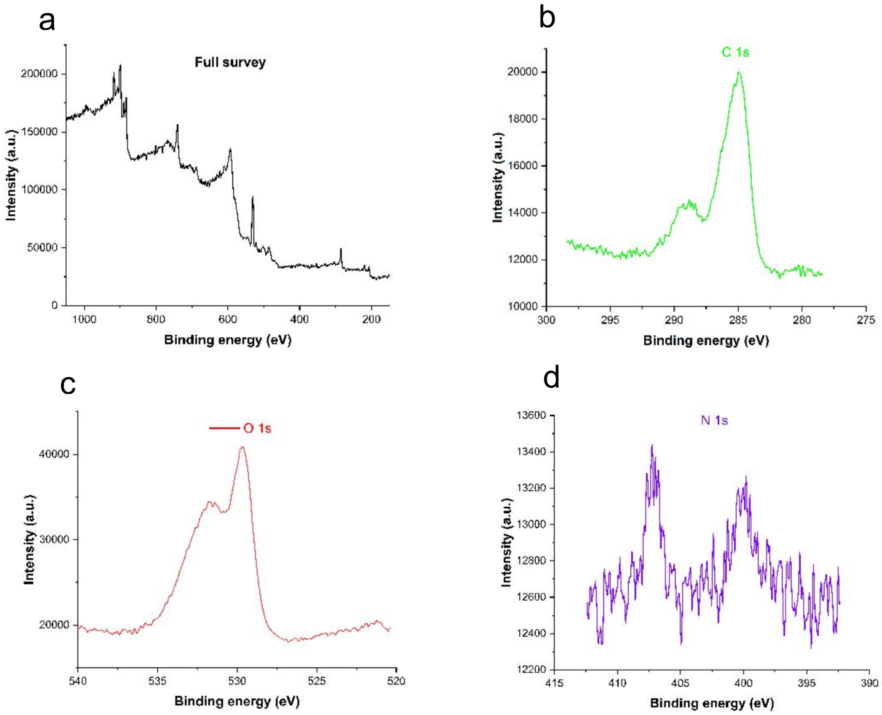

Figure S1. Full survey of D-CeO_2 (a) and fine scan of C (b), O (c), and N (d) elements.

Table S1- Ce 3d spectra peaks of D-CeO_2 and Ce^{4+}/Ce^{3+} ratio calculation.

**Ion**	**State**	**Binding energy (eV)**	**Ratio (%)**
	V	882.9	18.49
	U	901.1	16.76
**Ce^{4+}**	V''	889.9	8.23
	U''	908	8.51
	V'''	898.4	13.11
	U'''	917	13.58
	V_0	881.6	1.08
**Ce^{3+}**	U_0	899.3	1.11
	V'	886.5	9.41
	U'	904	9.72
**Ce^{4+} (%)**	78.68		
**Ce^{3+} (%)**	21.32		
**Ce^{4+}/Ce^{3+}**	3.69		

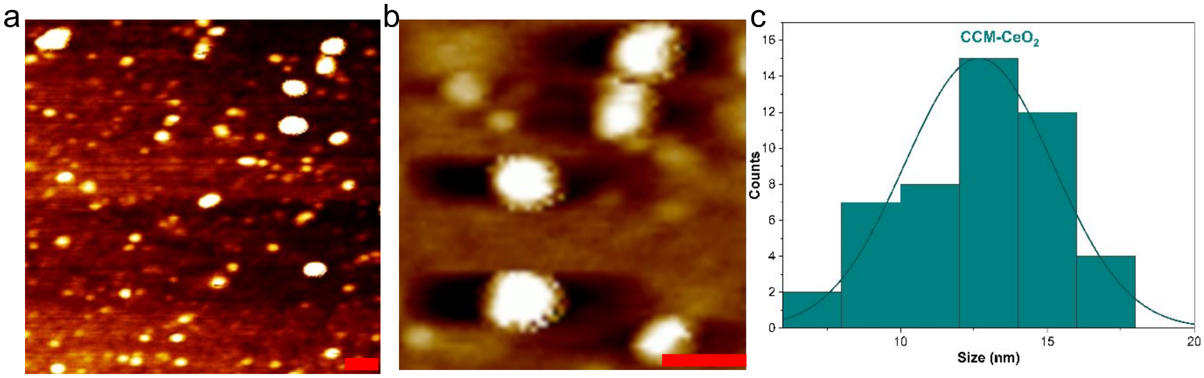

Figure S2. AFM images of CCM-CeO_2 (a), enlarged images of CCM-CeO_2 (b), and its size distribution (c). Scale bar: 25 nm.
